# Sex and Gender Differences in Prevention of Type 2 Diabetes

**DOI:** 10.3389/fendo.2018.00220

**Published:** 2018-05-04

**Authors:** Jürgen Harreiter, Alexandra Kautzky-Willer

**Affiliations:** Gender Medicine Unit, Division of Endocrinology and Metabolism, Department of Internal Medicine III, Medical University of Vienna, Vienna, Austria

**Keywords:** sex, gender, type 2 diabetes, obesity, prevention, bariatric surgery, sex hormones, ethnicity

## Abstract

Lifestyle intervention programs are effective in the prevention of type 2 diabetes mellitus (T2DM) in high risk populations. However, most studies only give limited information about the influence of sex and/or gender effectiveness of these interventions. So far, similar outcome was reported for diabetes progression and weight loss. Nevertheless, long-term data on cardiovascular outcome are sparse but favoring women regarding all-cause and cardiovascular mortality. In both men and women, sex hormone imbalances and reproductive disorders are associated with a higher risk of T2DM development. Diabetes prevention approaches are reported for polycystic ovary syndrome, gestational diabetes mellitus, and erectile dysfunction and are presented in this review. In the surgical treatment options for morbid obese patients, sex and gender differences are present. Choices and preferences of adherence to lifestyle and pharmacological interventions, expectations, treatment effects, and complications are influenced by sex or gender. In general, bariatric surgery is performed more often in women seeking medical/surgical help to lose weight. Men are older and have higher comorbidities and mortality rates and worse follow-up outcome after bariatric surgery. A more gender-sensitive clinical approach, as well as consideration of ethnicity may improve quality of life and increase health and life expectancy in men and women with a high risk for subsequent progression to T2DM.

## Introduction

The number of patients affected by type 2 diabetes mellitus (T2DM) is increasing worldwide. In 2015, around 415 million people were affected and an expected number of nearly 650 million subjects in 2040 was estimated (1). T2DM is an increasing problem with immense importance on the health care systems worldwide such as on the affected individuals. To reduce this tremendous increase successful prevention methods are necessary, which can be implemented in a population wide setting (2). These approaches could potentially reduce the number of diabetic cases in the future considerably (3, 4). Many trials have effectively tested different lifestyle and pharmacological intervention methods, but did not consider sex or ethnicity as a relevant factor (5). However, several aspects of sex or gender were identified as risk factors for T2DM, such as pathophysiology, onset age, detection, burden, or management of T2DM (5–7). Moreover, the diabetes risk assessment tool of the ADA guidelines includes sex-specific items and male sex is acknowledged as diabetes risk factor (8). In the same guidelines, high-risk populations for the development of T2DM, such as African American, Hispanic, Native American, Asian American and Pacific Islanders, need to be considered for early prediabetes/diabetes screening (8). Indeed, diabetes prevalence is lowest in the non-Hispanic white population compared to all other ethnicities (9). In Asian populations, diabetes screenings are recommended to begin at lower BMI’s than in other ethnicities (23 vs 25 kg/m^2^), due to differences in body composition (higher amount of visceral fat), higher insulin resistance, and lower second phase insulin secretion (10). Men are marginally higher affected by diabetes all over the world; they are diagnosed at younger age and at lower levels of overweight/obesity.

In weight loss studies, the majority of participants are female. Women might be more concerned by increased or increasing weight and show greater weight control involvement and thus seek medical help for weight loss therapy/surgery earlier (11, 12). Greatest effectiveness was seen in morbidly obese patients following bariatric surgery—usually with high female proportion—leading to impressive weight loss and a relative risk reduction of the diabetes incidence of up to 84% (13, 14). Lifestyle interventions effectively reduced diabetes risk by almost 60% (13). At present, there is no physiological explanation for major sex differences with regards to weight loss (15), but some trials indicate that women lose less weight than males including the more harmful visceral fat mass (15). Interestingly, women with T2DM, but not men, have a different perception of their body image compared to healthy women. They have significantly higher BMIs, at the same body image figure, than women without T2DM (16).

Although the sex of a patient has multiple effects on various aspects in T2DM management, these aspects however currently have no impact on clinical decisions. Therefore, this review briefly addresses and summarizes the important issues of sex and gender differences as well as ethnical aspects in the prevention of T2DM.

## Methods

A critical review of available peer-reviewed literature in medical databases was conducted. The search terms included the terms prevention, preventive measures, intervention, physical activity, healthy diet, T2DM, sex and/or gender, male, female, obesity, bariatric/metabolic surgery, polycystic ovary syndrome (PCOS), erectile dysfunction (ED), gestational diabetes mellitus (GDM), ethnicity, and race. Medical Subject Heading (MEsH) search terms were used if available and search terms were connected with Boolean terms. Only articles reporting human data and not older than 15 years were included. Older material was considered if highly relevant for this review or general understanding. Relevant literature was carefully examined and an additional hand search was performed to identify further relevant literature from the reference lists. A narrative synthesis approach describing results descriptively and not systematically was chosen in view of heterogeneous and limited information material.

Sex differences describe biological differences between women and men. Differences in sex chromosomes, sex-specific gene expression of autosomes, sex hormones, and their influence on organ systems are responsible for these differences between men and women (6). Gender differences are a result of sociocultural processes. Those processes specifically relevant for gender differences comprise differences in behavior, environmental exposition to specific influences, nutrition, life style and stress or different attitudes toward treatment and prevention (6). An accurate distinction between “sex” and “gender” effects is often not possible because these complex processes are interrelated and interact with each other during lifetime, including epigenetic mechanisms. Therefore, in this review, sex will be used to indicate primarily biological differences and gender to describe predominant psychosocial influences. However, the manifold interactions between biological, societal, and cultural influences in the risk and potential prevention of T2DM have to be kept in mind.

## Interventions

### Lifestyle Changes

**Figure 1:**
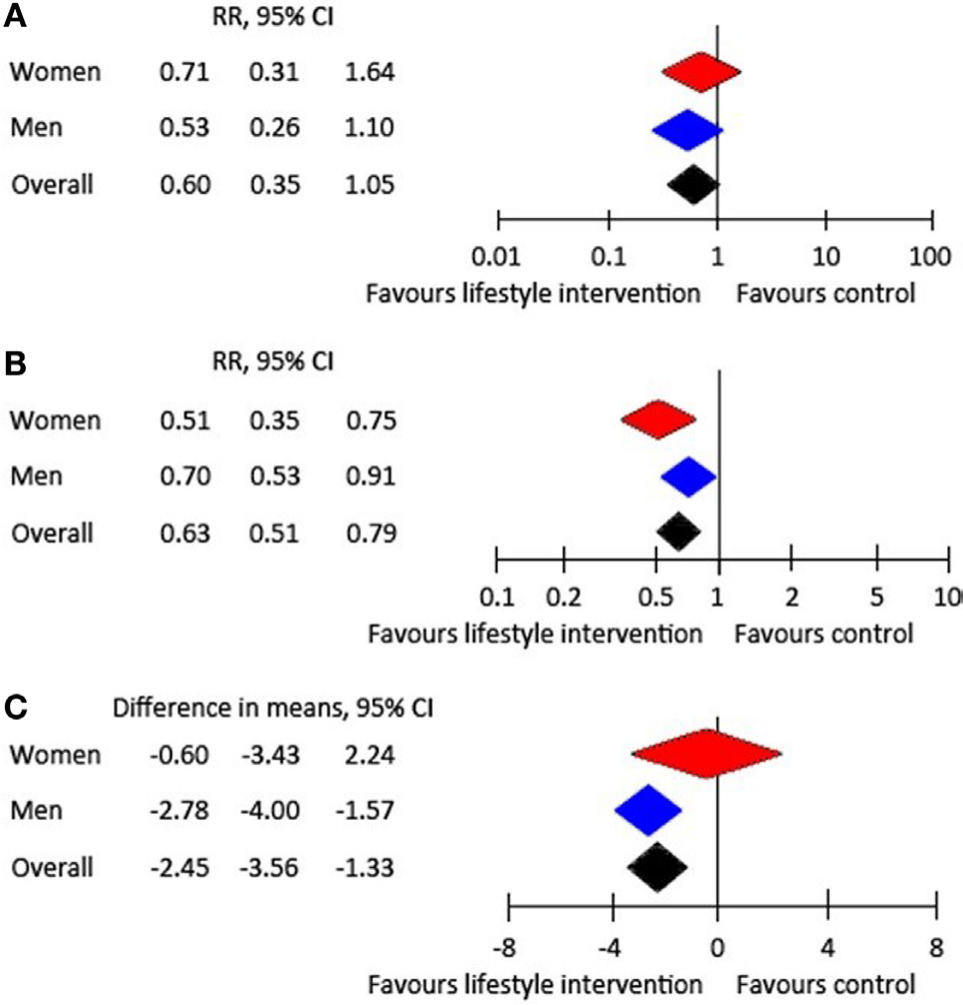
Effect of lifestyle intervention compared with treatment as usual: **(A)** RR of developing type 2 diabetes after 1 year, **(B)** RR of developing type 2 diabetes after 3 years, and **(C)** weight change after 3 years (5).

Few prevention trials and systematic reviews provide information on potential sex or gender differences in diabetes prevention. In men and women with pre-diabetes, a meta-analysis of diabetes-prevention trials showed equal effectiveness in diabetes risk reduction and weight loss in both sexes receiving lifestyle interventions, up to 3 years after intervention (5) (Figure 1). A nearly 40% risk reduction was observed after 1 and 3 years accompanied by a significant weight loss of nearly −2.5 kg after 3 years. Therefore, promotion of healthy behavior is important with special attention to those aspects of lifestyle changes that are more often neglected in one sex, which are healthy nutrition in men and physical activity in women (6, 11, 17, 18). Moreover, no sex differences in the association of oral glucose-lowering drugs with the reduction of T2DM were found due to limited data available (5). Unfortunately, information about relevant differences between men and women in adverse events was insufficient.

Interestingly, a recently published Indian study was able to demonstrate a stronger relative diabetes risk reduction in male and obese participants older than 50 years, who received a stepwise intervention approach initially starting with lifestyle changes and then followed by metformin prescription, if required (19). Men had a significant reduction of 37%, whereas women had a non-significant reduction in diabetes incidence of 24%. No significant differences between sexes were found, but men seem to achieve their physical activity goals after 6 months and 1 year significantly more often than women. Initially, women indicated to encounter more barriers to participate in the study (20). According to the authors, the latter had influence on recruitment outcomes and lifestyle changes. Overall, more than 63% of the study population was male.

In the Diabetes Prevention Program (DPP), intensive lifestyle modification reduced the diabetes risk in the intervention group (21). Although, women achieved significantly more lifestyle modification goals than men, the diabetes incidence was comparable. Baseline risk factor analysis revealed differences with higher age, waist circumference, fasting plasma glucose, caloric intake, blood pressure, and lower HDL cholesterol levels in men and higher BMI and physical inactivity in women. In patients with weight loss below 3%, no sex differences were observed after 1 year. Weight loss from 3 to 7% led to lower 2 h glucose and insulin levels, as well as improved insulin resistance in men compared to women—above 7% additionally ameliorated triglyceride and HbA1c levels were reported, which was more pronounced in men (21).

Recently, a study examined activity and sedentary time in participants of the Diabetes Prevention Program Outcome Study (DPPOS) followed-up 10 years after the lifestyle modifications in the original trial. Higher moderate to vigorous physical activity (MVPA) was assessed by accelerometers in all DPPOS subgroups with T2DM or IGT in both sexes, compared to adults participating in the National Health and Nutrition Examination Survey (NHANES) (22). This was especially pronounced between 60 and 69 years in men and women with T2DM and men with IGT. In general, MVPA was significantly higher in men of the DPPOS follow-up compared to women and women more often had low physical activity levels. Interestingly, no differences in sedentary behavior were found between sexes. Physical activity decreased significantly with increasing age. Nevertheless, the evaluation of longitudinal questionnaire data found significantly increased activity in all groups in the DPPOS compared to baseline data of the original DPP trial, which demonstrates lasting lifestyle changes in men and women of all age groups, following effective lifestyle intervention programs and is in contrast to reports of stagnant or decreasing physical activity levels (22–24).

In women participating in the lifestyle intervention group of the Da Qing Study, a potential benefit was shown in cardiovascular and all-cause mortality (25). After 23 years of follow-up, the female risk of cardiovascular mortality and all-cause mortality was reduced by more than 70 and 50%, respectively, in the intervention group compared to usual care, whereas in men, no intervention effect was found. Part of this might be explained by a high prevalence of smoking in men compared to low prevalence in women. However, this is only speculative and was not extensively discussed in the original paper. Furthermore, low participant numbers were followed up after this long-time period and different lifestyle behavior, cultural and ethnical differences need to be taken into account before a generalization of these results is possible at all. To further clarify potential sex or gender differences as well the influence of ethnicity and other sociocultural aspects in mortality after lifestyle intervention more evidence from long-term follow-up prevention trials is necessary.

As described above, ethnical differences next to sex and gender differences need to be considered in weight loss and lifestyle intervention programs. A secondary analysis of the DPP demonstrated significantly lower success in weight loss in the lifestyle intervention arm in black women compared to all other ethnical groups (26).

Latinos appeared to benefit less from the NDPP with lower weight loss most likely because of a 50% lower attendance rate of the programm sessions (27). In addition this ethnic group was younger, had a lower income and comprised an even higher percentage of women compared to the Non-Hispanic white participants (27). Barriers to attendance among underserved patients might be the main reasons but were not further investigated.

Sex differences were also found in the Prevention of Diabetes and Obesity in South Asians (PODOSA) study between Pakistani and Indian male and female migrants in a secondary analysis of this weight loss intervention study. Asian men in the intervention arm (culturally adapted diet and physical activity) had higher weight loss compared to Asian women (28). This finding was corroborated by a study investigating lifestyle effects on Asian Indian migrants older than 50 years. Asian Indian men had significant reductions in body weight, body fat, waist circumference, and abdominal fat, whereas in women, these changes were not observed after 5 months of intervention (29).

Culturally tailored DPPs were shown to be effective in another recent randomized control trial, including Asian Indians (Gujarati) living in an urban community (30). After a 12-week group-based lifestyle intervention program aiming for weight loss and increased physical activity, the intervention group had significantly lower weight, HbA1c, and waist circumference as well as significantly increased physical activity compared to the control group. Sex differences were not analyzed. A systematic review identified 34 culturally adapted diabetes prevention intervention trials specific for minority populations of which 25 trials significantly improved HbA1c, fasting glucose, or weight loss (31). A framework of four key domains including Facilitating Interventions through Language, Location, and Message (FiLLM) was developed to assess overall effectiveness of these. In this context, six mediators were identified, which are relevant for successful lifestyle behavioral change, which are gender, generation, geography, genes, religion, and gaps in knowledge and economic resources (32). All these studies demonstrate the necessity for awareness of the high risk among minority groups and for evaluation of culturally tailored interventions based on sociocultural and environmental differences in future DPPs. Analysis by sex is often missing in studies in minorities and should therefore also be included in future study designs.

As discussed in a recent article, lifestyle intervention compared to no intervention is a cost-effective strategy (33). The potential cost effectiveness of hypothetical prevention methods in men and women at all ages compared to no intervention was reported. A Swedish setting was used in the analysis, assuming a hypothetical intervention—comparable to those used in the Finish Diabetes Prevention Study—including male and female sex, different ages, and several states of impaired glucose tolerance (33). A high probability of cost effectiveness was shown, with an assumed threshold of 50,000 Euros per QALY gained. The authors concluded that a DPS-like intervention is highly likely to be effective in reducing risk of T2DM progression and thus will be cost-effective in both sexes, among different glucose tolerance stages and across all age categories.

Key points: data regarding sex and gender differences of diabetes prevention trials are limited. Comparable improvements in glycemic control or weight loss up to 3 years after intervention were reported in men and women. However, various sex/gender as well as ethnical differences regarding effectiveness, lifestyle goal achievement, or barriers to participate in a study were indicated. Lower cardiovascular mortality in women participating in the lifestyle arm and no effect in men was observed compared to controls (Table 1).

**Table 1:** Summary of sex and gender differences in diabetes prevention.

	Men	Women	Notes	Reference
Lifestyle changes	+	+	RCTs: after 3 years equal effectiveness in both sexes: 40% risk reduction and significant weight loss; no sex differences in the association of oral glucose-lowering drugs with T2DM risk reduction	(5)
	+	(+)	Indian RCT: stronger diabetes risk reduction in elderly obese men, more barriers to participate in women	(19, 20)
	+	(+)	DPP and DPPOS: greater improvement in glucose tolerance, insulin resistance, HbA1c, and triglycerides in men. Higher MVPA in men, more often low physical activity in women. Black women lower success in weight loss	(21, 22, 26)
	0	+	DAQING: lower cardiovascular mortality and all-cause mortality in women in long-term follow-up	(25)

Weight management programs	+	(+)	Men are more successful in reducing and maintaining weight than women in the majority of studies. Race-sex subgroup analysis: black and white men more success in weight loss than women	(11, 34, 37)
	(+)	+	Women are more successful in reducing and maintaining weight with pharmacological approaches (Orlistat)	(11, 34)

Exercise Program	+	+	Both sexes prefer to exercise with members of their own sex relative to exercising in gender-mixed groups	(42)
	+	+	Sex-specific team-sports-based intervention programs in men and women show higher effectiveness, weight loss, amelioration of blood pressure, and quality of life compared to controls	(51)

Bariatric Surgery	+	++	Strong diabetes risk reduction: higher frequency of bariatric surgery in women due to differences regarding expectations and willingness, related to body image and psychosocial factors in women or obesity-related health concerns and morbidities in men	(14, 59, 63)
	−	−	Higher complication and mortality rates following surgery in men, higher rates of revision procedures in women	(60, 61, 65)
	+	++	Gastric banding may be less efficient in males than females	(69)

Hormonal imbalance	−	−	Increased diabetes risk in men with hypogonadism or women with hyperandrogenemia	(78)

Erectile dysfunction	+	n.a.	Positive effect of lifestyle intervention on erectile function, weight loss, and physical activity; reduction in all-cause mortality, trend for reduction of major adverse cardiovascular events or incident diabetes	(91, 98)

Hormone replacement therapy	+	n.a.	TRT: improvement in anthropometrics, glucose tolerance, lipids, blood pressure, and sexual function and desire	(89)
	n.a.	+	HRT after menopause reduces the risk of T2DM and improves glycemic control. HRT within 10 years after menopause and below 60 years of age is effective in preventing development of T2DM and cardiovascular disease	(118)

PCOS	n.a.	+	Lifestyle intervention and metformin effectively reduce weight and subcutaneous fat	(109)
	n.a.	+	Myoinositol supplementation—lower insulin resistance and a trend for reduction of androgens	(110)
	n.a.	+	DPP4 inhibitors—improvements in beta cell function and insulin resistance with lower conversion rates to T2DM	(111)

*0, no effect; + positive effect; −, negative effec; n.a., non appropriate; RCT, randomized controlled trial; DPP, Diabetes Prevention Program; DPPOS, Diabetes Prevention Study, Diabetes Prevention Study Follow Up; MVPA, moderate to vigorous physical activity; ED, erectile dysfunction; TRT, testosterone replacement therapy; HRT, hormone replacement therapy; PCOS, polycystic ovary syndrome; DPP4, dipeptidylpeptidase 4*.

### Weight Management Programs

A systematic review of RCTs with long-term weight management interventions found that men are less likely to participate in such interventions, representing about one-third of the total study population (34). Nonetheless, attrition rates are much higher in women included in a trial and thus men are more likely to complete a study. In this analysis, including more than 8,400 male and female participants of 13 RCTs, no significant differences in weight loss or percentage loss between men and women were observed; however, strategies how to reduce weight effectively may differ between sexes. Men may have higher benefit of a low-fat diet and defined physical activity interventions with individual and tailored support. In contrast pharmacological approaches, as shown for Orlistat, are more successful in reducing and maintaining weight in women. Social support from a partner is reported to be more helpful in men. The authors conclude that interventions need to be developed, which attract men and make them feel convenient, which is probably best delivered by gender-sensitized interventions (11, 34).

An Internet delivered weight-loss intervention found significantly higher weight reductions in non-Hispanic white women compared with African American women, which was explained by higher website engagement and changes in eating behavior in non-Hispanic white women (35). Interestingly, contrary to African American women maintaining lifetime normal weight, those who were overweight before in their lives were more insulin sensitive and thus might be predisposed for obesity and fat accumulation (36). An ethnicity specific effect of higher insulin responsiveness was identified in African American women compared with European American women, which might be further relevant for obesity predisposition.

A study trying to identify predictors of successful weight loss and maintenance (WLM) in race—sex subgroups (black or white men and women) found within race, men losing significantly more weight than women, but no differences in weight loss within sex group between races (37). Predictors beyond initial weight loss identified in most subgroups were healthy nutrition at entry and improvements in diet throughout the study period. However, black men showed no potentially modifiable variables except initial weight loss. In white men, only MVPA additionally predicted long-term weight loss. Further important factors for successful weight loss identified were social support and social pressure for weight loss activities, which were higher in white compared to black women (38). Social support seems to be an essential motivator, especially in black women, to participate successfully and engage more intensively in weight loss activities (39). In a study investigating the effectiveness of a community-based weight loss program with implementation of two different strategies over a duration of 2 years in an obese, low-income Latino population, women had significantly lower weight loss than men (40). However, sustained weight could not be achieved in either sex.

Key points: men are less likely to participate in weight management interventions than women. Weight loss is more successful in black and white men compared to women. Pharmacological approaches are more successful in reducing weight in women. Social support from a partner is more helpful in men (Table 1).

### Exercise/Sports Programs

In the analysis of the total study population of the Henry Ford Exercise Testing (FIT) project, including men and women, a reduction of risk for incident T2DM by 54% was found in those participants with at least 12 metabolic equivalents compared to those subjects achieving less than 6 METs regardless of sex (41).

Based on recent evidence both sexes prefer to exercise with members of their own sex relative to exercising in gender-mixed groups (42). These preferences for gender-segregated physical activity groups are even stronger in overweight subjects. This was corroborated by another study, which reported similar observations in obese adults (43). Additionally, obese participants indicated to prefer activities with people of similar age. So far this was only reported in observational studies and needs further proof in randomized controlled trials. The Canadian GOAL study will try to investigate age- and gender influences on physical activity adherence behaviors in three different age and sex groups: one group consists of participants of similar age and same sex, the second group includes same sex but different ages and the third group is a group of patients of different sex and ages (44). Overweight and obese participants of physical activity classes might feel embarrassed or stigmatized, emotionally insecure or unaccepted and thus seek solidarity and empathy in same age same sex physical activity groups (43). Therefore, same sex-group-based exercise programs may be more successful, probably due to better motivation, adherence behavior, and social congruence. Shared social identity, which can be described as the identification of a person with a group with similar characteristics (e.g., sex, age, physical appearance, ethnicity, cultural background) and the motivation to get involved, integrate and become a member of this group, seems a major motivational factor for participants of group-based exercise programs to show higher adherence and thus potential better outcome than members feeling different from the group (45, 46). This strategy seems to provide a dynamic body for the development of new prevention programs; however, the social identity approach is currently under-investigated and further evidence is necessary to prove effectiveness and feasibility of such interventions (45).

In fact, a gender-sensitized weight loss and healthy living program based on a social identity approach for overweight and obese men delivered by football clubs was very successful in Scotland (47). In this pragmatic RCT, the intervention group lost almost 5 kg more weight than the comparison group within 1 year. In the intervention group, blood pressure and percent fat content decreased significantly and diet and quality of life improved. Such male-specific initiatives may be very promising in tackling male obesity, as men are under-represented in common gender-integrated weight loss programs. Currently, more gender-sensitized studies are ongoing, which are evaluating the effectiveness and cost-effectiveness of an evidence and theory-based, gender-sensitized, multinational health program attracting men through the loyalty to their football clubs in four European countries (48). Another program tried to improve physical activity and healthy eating choices, leading to subsequent weight loss and other health outcomes through a lifestyle program over 52 weeks (12 weeks active phase and 40-week minimal support phase) for overweight or obese male hockey fans with BMI above 28 kg/m^2^ in Ontario, Canada (49, 50). After 12 weeks, the intervention group had 3.6 kg higher weight loss compared to control group, which was preserved until the end of the study after 12 months. Moreover, increases in physical activity, self-rated health, a healthier diet, and improvements in several other clinical parameters were observed. A further gender-sensitized hockey intervention program based in Canada called HATTRICK, which is currently ongoing, is also focusing on improving physical activity and healthy eating in inactive and overweight men and will report further evidence on gender-sensitized lifestyle intervention provided to inactive men through team-sports-based intervention programs (51). These studies are aimed to address overweight or obese subjects and not directly to prevent T2DM progression, although excess weight is a substantial risk factor for insulin resistance and diabetes progression. So far, these programs are undoubtedly wonderful examples how to promote physical activity and lifestyle changes in highly inactive men at risk.

A recent Swedish cohort study with military conscripts reported that a combination of low aerobic capacity and low muscle strength at the age of 18 years was associated with a threefold higher risk of T2DM in adulthood, which was independent of BMI, family history, or socioeconomic factors (52). This study demonstrates, that early prevention starting at young age, which improves aerobic and muscular fitness, was able to reduce T2DM in adulthood in male subjects independent of body weight. However, the authors concluded that more research with longitudinal fitness measurements is needed to find out important windows of susceptibility (52). This is corroborated by another huge nationwide cohort study from Israel, which investigated the association of childhood obesity at the age of 17 years, with diabetes mortality followed over a median time period of 18.4 years. Increased diabetes mortality starting already at normal weight from the 25th–49th BMI percentile and an 8- and 17-fold higher risk for diabetes mortality in overweight and obese subjects, respectively, after adjustment for sex, age, birth year, height, and sociodemographic variables was found (53). Mortality rates increased tremendously with increasing age. Further risk factors identified were lower body height, low socioeconomic status, or educational status, North African and former Union of Soviet Socialist Republics origin, as well as male sex.

Gender-sensitized intervention programs were also studied in women and demonstrated promising results in the reduction of various risk factors of cardiovascular and metabolic disease (54), as well as bone metabolism and cancer risk. A recently performed Scandinavian study was able to demonstrate the effectiveness of soccer training in middle-aged women with mild hypertensive disorder and reported an amelioration of blood pressure, plasma lipid levels, bone density, total body fat mass and better performances in endurance and sprint compared with control (55). This study, among others, clearly shows that team-sports-based interventions are also an effective tool to improve cardiovascular and metabolic health in untrained middle-aged women (54, 55). Similar beneficial effects of soccer training in women on cardiometabolic parameters were reported earlier by the same group of investigators (56). A further study compared soccer training with different intensities of swim training and control in middle-aged women and found higher bone turnover markers after 15 weeks of soccer training, whereas no changes were found in swim groups and control (57). High intensity intermittent training was reported to be associated with improvements in metabolic parameters and glycemic control and team-sports-based interventions incorporate a blend of various training components, as endurance, high-intensity exercise and resistance training (54). Therefore, such trainings and broad-spectrum health improvements may also be applicable in women with prediabetes or high risk to develop T2DM. However, studies to investigate team-sports-based interventions in the prevention of T2DM in women are missing. Studies including women with T2DM applying team-based soccer and dietary interventions for 12 weeks demonstrated increased insulin sensitivity and ameliorated lipid profiles, as well as higher maximal oxygen uptake in women with T2DM in the soccer and dietary intervention groups compared with dietary group alone (58). Team-based soccer activities are popular in Scandinavian countries and give evidence that a social identity approach is successful to motivate inactive women for participation in team-based soccer training as shown in a Danish initiative called “Football Fitness” and a similar project on Faroe Islands (54). They truly might have the potential to reduce T2DM progression at a nationwide scale in women (and men) at risk, although this needs further proof.

Key points: physical exercise reduces the risk for incident T2DM in men and women. Sports-clubs-based, gender-sensitized lifestyle programs for men and women demonstrated significant weight reduction and improvement in cardiometabolic parameters. Socially and culturally tailored intervention programs are able to increase adherence and motivation to participate and thus have the potential to increase the outcome of such interventions (Table 1).

### Bariatric Surgery

In morbidly obese patients, lifestyle intervention or pharmacological intervention do often not result in significant and sustained weight loss. Thus, surgical options are used, which have demonstrated a significant potential in reducing weight. In literature, several sex and gender differences are reported regarding expectations and willingness toward surgical treatment, complication rate and weight reduction.

A systematic review showed that females have greater willingness to undergo bariatric surgery (14). Female patients have higher expectations regarding weight loss and reasons for undergoing surgery are strongly related to body image and psychosocial factors. Males report obesity-related health concerns like diabetes, hypertension, or sleep apnea, as crucial for their decision (59).

Indeed, male patients suffer more comorbidities before a surgical intervention and have higher complication and mortality rates following surgery (60, 61). Also, other studies corroborate these differences in clinical aspects between men and women before laparoscopic RYGB. In a large observational study including more than 83,000 patients (62) with the majority being female, men were older and had significantly higher rates of metabolic impairment (diabetes, gout, dyslipidemia), abdominal hernia, liver disease, alcohol and tobacco abuse and cardiopulmonary disease except asthma, which was more common in women, next to gastroesophageal reflux, cholelithiasis, abdominal panniculitis, back and musculoskeletal pain, and mental health disorders. In a German registry, female patients are younger and present with lower BMI than men (63). Males also showed higher prevalence of comorbidities, such as hypertension, T2DM, with and without insulin therapy, and sleep apnea, as well as complication rates (63). A meta-analysis demonstrated that mortality within 1 month after surgery was 0.1% in females but 4.7% in males (61).

Comparing patients selected for sleeve gastrectomy with laparoscopic Roux-en-Y gastric bypass (RYGB) revealed that female sex, gastroesophageal reflux disease, and an age of the surgeon above 40 years, were associated with greater probability of undergoing RYGB procedure (64). Higher BMI, presence of T2DM, as well as undergoing sleeve gastrectomy were associated with less weight loss. In general, complications include higher leakage and sepsis rates following RYGB or sleeve gastrectomy in males than females, but post-operative complications depend on age and BMI. Interestingly, a recent Australian study found that 27% of all bariatric procedures are revisions causing a huge socioeconomic burden. More than 80% of these revisions were performed in females (65). A prediction tool for postoperative complications after bariatric surgery includes six parameters of which gender (Odds ratio 1.4) is one of them, to identify low to high risk patients (66). The authors found in their multivariable analysis that next to male sex, the use of anticoagulants (OR 1.5), COPD (OR 2.3), dyslipidemia (OR 1.4), history of psychiatric disease (OR 1.3), and revision procedures (OR 1.5) are the other relevant predictors.

Severe obesity is often associated with hormonal imbalance. In a systematic review, gonadal dysfunction was observed in 36% of women featuring PCOS and 64% of men featuring male obesity-associated secondary hypogonadism (MOSH) (67). After bariatric surgery, this resolved in 96% of women and 87% of men, respectively, accompanied by increases of SHBG in both sexes and testosterone in men and decreases of estradiol in both sexes and testosterone in women, respectively. Furthermore, women showed an amelioration of menstrual dysfunction and typical signs of androgen excess as hirsutism. In a longitudinal study of obese patients with obstructive sleep apnea undergoing RYGB procedures, improvement of sleep parameters, obesity indices and metabolic outcomes were of similar magnitude in both sexes, except for triglycerides, which improved more in men than women (68). In another study, using gastric banding procedures 65% of the female compared to only 54% of the male diabetics had complete diabetes remission within 5 years after surgery and women also showed higher remission for hypertension and sleep apnea (69). Gastric banding may be less efficient in males than females, compared with other bariatric procedures, but at present there are no gender-tailored bariatric strategies.

Follow-up visits after bariatric surgery are essential to identify and prevent malnutrition and complications. A recent French retrospective study, including 16,620 patients who underwent bariatric surgery since 2009, found in a multivariable analysis that male sex, young age, no diagnosis of T2DM and poor 1 year follow-up was a predictor of poor 5-year follow-up (70). In studies with follow-up until 10 years after bariatric surgery, male sex was associated with an unfavorable energy and macronutrient intake and higher alcohol intake, which was also shown for younger age and sedentary behavior before surgery (71). Furthermore, male sex was associated with higher weight regain after RYGB procedure, next to other factors as white ethnicity and higher socioeconomic status (72). These studies demonstrate that men are at an increased risk for unhealthy behavior after a bariatric procedure, but also have higher mortality and complication rates, which is probably related to higher age, BMI, and an unhealthier condition already before surgery. Nevertheless, women have higher rates of revision procedures. Higher awareness of greater risk of the male population after bariatric surgery is needed to improve health outcome and probably more guidance is needed after bariatric surgery for male and female subjects at high risk for complications. More studies will help to further understand sex and gender differences after bariatric procedures.

Ethnical differences were observed in weight loss after bariatric surgery with African Americans losing less weight after RYGB and gastric banding than Caucasian patients (73). However, these differences were not explained by demographic, clinical, or behavioral characteristics. Further studies corroborated these findings and found lower weight loss after RYGB, sleeve gastrectomy, and gastric banding in African American compared with Hispanic and non-Hispanic white patients (74, 75). However, these differences did not influence the remission of co-morbidities (76).

In a study investigating race and gender effects bariatric surgery, significant higher weight loss after RYGB in non-Hispanic black man compared with non-Hispanic white men was found, whereas in women no such differences were reported. Ethnicity and sex were among the strongest predictors for weight loss next to age, weight at time of surgery, diet soda, and water intake (77).

Key points: bariatric surgery is very effective in weight reduction and thus prevention of T2DM. Women undergo bariatric surgery more often and show higher willingness to perform bariatric surgery. Male patients suffer more comorbidities before a surgical intervention and have higher complications and mortality rates after a surgery, women have higher revision rates. Men are at an increased risk for unhealthy behavior after a bariatric procedure. In African Americans, lower weight loss was observed (Table 1).

## Reproduction/Sex Hormones-Related High-Risk Groups and Interventions

Reproductive disturbances may help to identify subjects at risk in both sexes. Interestingly, a meta-analysis demonstrated the effects of sex hormones in men and women and revealed higher T2DM risk in subjects with sex hormone imbalance (78). Men with low testosterone levels had higher risk for T2DM, whereas in women associations between high testosterone levels and high diabetes risk were found. Among T2DM patients, men indicated a significantly higher sexual dissatisfaction compared to women and were in higher need for care in a Dutch study (79). ED was found in two-third of the men, whereas half of the women indicated low sexual desire and lubrication problems, respectively. In another study, a higher association of sexual dysfunction with depression was found in patients with T2DM in both sexes, particularly at higher age (80). A secondary analysis of the DPP found that reductions in visceral or subcutaneous adipose tissue were associated with significantly higher testosterone levels in men and SHBG levels in both sexes, which was independent of ethnicity (81).

### Men/Male-Specific Risk

Low testosterone levels and/or ED may characterize male-specific high risk for T2DM, obesity, and the MEtSy (6, 82). In addition, poor glycemic control is associated with increasing risk for ED (83). A faster disease progression was reported in men with T2DM, with an ED diagnosis more than a decade earlier (84). Thus, ED may serve as an indicator for undetected impaired glucose tolerance. In patients with T2DM, systemic endothelial dysfunction and low-grade inflammation might be an underlying mechanism for ED and subsequent macrovascular complications (83, 85, 86). In addition, hypogonadism is strongly associated with ED *via* direct and indirect metabolic effects and testosterone replacement might have positive effects on various regulators and mediators involved in erectile function (86, 87). A very recent systematic review investigating testosterone replacement therapy (TRT) in men suffering from hypogonadism found four trials which reported significantly better erectile function, sexual satisfaction, and higher sexual desire and libido, with no effects on mood or energy (88). A further review found improvements in components of the metabolic syndrome such as decreases in weight, BMI, waist circumference, glucose levels, HbA1c, cholesterol, and blood pressure in hypogonadal men receiving TRT, but also reporting conflicting literature (89). Thus, studies are needed to further evaluate the efficacy of TRT in hypogonadal men and in particular in the prevention of T2DM.

In general, lifestyle changes with improvement of healthy diet and physical activity, weight loss, the management of comorbidities, and good glycemic control are recommended for men with ED and T2DM (90). The positive effect of lifestyle intervention on erectile function, weight loss, and physical activity was shown in a *post hoc* analysis of middle-aged, overweight/obese men with T2DM in the LOOK AHEAD study (91). After 1 year in the intervention group, a significant improvement in erectile function, next to weight loss and increased physical activity, was found. A systematic review of 6 clinical trials, with 740 men with ED, reported an improvement of sexual function after at least 6 weeks of lifestyle or pharmacological intervention for cardiovascular risk factors (92). This was corroborated by a Japanese observational study, which found an inverse association of physical activity level with severity of ED in men with T2DM (93). Also, positive effects of lipid lowering compounds like statins were confirmed in men with ED (94), which is contrary to reports about subsequent ED as an adverse effect of statin therapy. A recent meta-analysis did not find any association between statin use and new onset ED (95). Several studies have shown that weight loss, increase of physical activity, smoking and alcohol cessation, as well as stress reduction, are important prevention strategies to tackle sexual dysfunction and other health implications (96). So far, few studies examined the effect of physical fitness in men with ED on health outcomes. In the DPP intensive lifestyle intervention was associated with increased testosterone levels, whereas these changes were not found with metformin or placebo. However, testosterone increases were not associated with significant changes in mood in the lifestyle intervention or placebo arms, but significantly improved depressive symptoms were found with increasing testosterone levels in the metformin arm (97).

In a study examining men with pharmacologically treated ED from the Henry Ford Exercise Testing (FIT) project, the associations of exercise capacity with cardiovascular and metabolic outcomes were investigated (98). A reduction of 16% in all-cause mortality with increasing fitness and non-significant reductions for major adverse cardiovascular events or incident diabetes were identified. A window of opportunity of up to 5 years was discussed from the onset of ED to a symptomatic cardiovascular event (99). Thus, early introduced preventive approaches seem to be important to improve ED and avoid cardiometabolic disease progression. However, limited evidence exits about lifestyle intervention in men with ED and subsequent disease progression, and thus, further studies are needed.

### Women/Female-Specific Risk

In women, reproductive factors and reproductive history help to identify women at higher risk for T2DM. Early menarche, irregular cycles, higher androgen levels or PCOS, and most of all a history of gestational diabetes (GDM), affecting approximately 15% of pregnant women, are related to higher diabetes risk (6, 100). The EPIC-InterAct study reported an association of early menarche between 8 and 11 years of age with 70% higher T2DM risk compared to menarche at 13 years of age (101). This was corroborated by a systematic review showing about 22% higher risk. A relation between higher prepubertal BMI and younger age of menarche onset is well-known; nonetheless in this study, an independent association between menarche and T2DM risk was shown (101). Older age at menarche did not decrease T2DM risk. Decreased insulin sensitivity and higher obesity risk in young female adults were also reported for early menarche, as well as higher risk for NAFLD (102–104), ectopic lipid accumulation, and GDM. Interestingly, application of metformin at age 8 years was able to delay menarche by more than 1 year and increased body height and reduced body weight, hepatic lipid accumulation and insulin, androgen and lipid levels in girls born with low body weight and precocious pubarche (105). Importantly, timing of metformin onset seems important; as in a further investigation, differences with higher prevalence of hirsutism, androgen excess, oligomenorrhea, and PCOS were found in those girls with later treatment start of metformin (106).

A higher susceptibility for metabolic disease and development of T2DM and GDM is well known in patients with PCOS with reported relative risks of about 2.0, and 3.9 with BMI above 30 for T2DM and 2.9 for GDM (107). Women with PCOS have higher prevalence of overweight/obesity including central obesity and BMI and show higher associations with increased insulin resistance and hyperinsulinism (108) compared with women without PCOS. Moreover, a genetic predisposition is widely discussed, which leads to polycystic ovary formation and finally to excess androgen production, LH production, and insulin resistance with hyperinsulinemia aggravated by excess weight and further stimulation of the ovarial theka cells to produce testosterone, which causes a plethora of variations in symptoms. Lifestyle intervention and change are very important in the prevention of T2DM for subjects at high risk as shown in several diabetes prevention trials (5). Large lifestyle intervention studies including PCOS women and testing diabetes prevention strategies are scarce. So far, lifestyle changes are reported to be beneficial and ameliorate fasting glucose (107). A meta-analysis provides evidence that in women with PCOS, a combination of lifestyle intervention and metformin is more effective in weight reduction and reduction of subcutaneous fat after 6 months of combined treatment compared to metformin alone or lifestyle and placebo groups, although no effects were documented on other metabolic parameters as insulin resistance, glucose, or lipid levels (109). Furthermore, higher probability of menstruation was reported in the lifestyle and metformin group. Another meta-analysis investigating the metabolic effects of myoinositol supplementation compared to control found beneficial effects of myoinositol with lower insulin resistance and insulin levels, as well as higher SHBG levels and a trend for reductions of androgens (110). However, the authors report huge heterogeneity of all included studies, which allows limited generalization, but plenty of space for new interesting research questions. Other promising new approaches including DPP4 inhibitors were tested over a 12-week period and have proven improvements in beta cell function and insulin resistance with lower conversion rates to T2DM (111) in the combined DPP4 inhibitor and lifestyle intervention group compared with lifestyle intervention alone. Nonetheless, this small pilot study included only 30 obese, metformin intolerant patients and thus these beneficial observations should not be over-interpreted as long as no further confirmatory studies and support these findings. Furthermore, GLP1 receptor agonists in obese PCOS women were successful in reducing weight and androgen levels but did not improve insulin sensitivity or menstrual irregularities (112).

Prevalence rates of obesity are increasing, which also affects women in childbearing age and represents a prominent risk factor of GDM (113). A cumulated GDM prevalence of 39% across the whole pregnancy was observed in obese pregnant women across Europe using IADPSG/WHO 2013 criteria (114). These women presented with a GDM prevalence of nearly 24% already before 20 weeks of gestation and a clustering of many factors of the MetSy (115).

Thus, evidence based prevention approaches in pregnancy to avoid GDM development (Table 2) and subsequent complications have to be found as women with a history of GDM have a 7.5-fold higher risk of T2DM development and bear a higher risk of consecutive cardiovascular disease (116, 117).

**Table 2:** Representation of GDM prevention trials (RCTs or meta-analysis of RCTs).

Population	Interventions	Results	Additional information	Reference
2,873 healthy pregnant women, low level of physical activity (exercising <20 min on <3 days per week), 1,434 intervention and 1,439 control group	Physical exercise programs that included low to moderate intensity exercises. No restrictions on frequency, duration, or type of training	Lower GDM risk (30% risk reduction) Lower maternal weight gain	GDM risk reduction and lower weight gain especially with physical activity program performed throughout pregnancy. No serious adverse effects	(119)

4,983 women and their babies	Combined diet and exercise interventions compared with standard care	No GDM risk reduction. No difference in maternal weight gain	Less preterm delivery; Comparable rates of cesarean section, LGA, stillbirth and neonatal death, shorter duration of hospital stay	(120)

132 women with BMI >25 kg/m^2^	Four-step multidisciplinary antenatal care (continuity of obstetric provider, regular weighing, nutritional and psychological advice) vs standard obstetric antenatal care	Lower incidence of GDM (83% less). Lower maternal weight gain	Comparable birth weight of newborns	(121)

2,152 pregnant women, BMI ≥ 25 kg/m^2^, 10–20 weeks of pregnancy	Early lifestyle intervention consisting of healthy eating advice and increasing physical activity compared to routine measures	No GDM risk reduction.^a^ No difference in maternal weight gain^a^	LGA not significantly different, less infants in intervention group with weight >4 kg, no differences in hypertension, pre-eclampsia, cesarean section, NICU admission, and hypoglycemia	(122)

1,555 pregnant women, BMI ≥ 30 kg/m^2^, 15–19 weeks of pregnancy	Behavioral intervention or standard antenatal care, once a week through eight health trainer-led sessions to endorse healthy eating	No GDM risk reduction.^a^ Lower maternal weight gain.^a^	LGA not significantly different, no significant differences in adverse birth outcomes. Increase in physical activity, reduction in dietary glycemic load, and maternal sum of skinfold thickness	(123)

269 pregnant women, history of GDM or BMI ≥ 30 kg/m^2^, before 20 weeks gestation	Individualized combined lifestyle intervention, focus on diet, physical activity, and weight control	Lower GDM risk (39% risk reduction). Lower maternal weight gain	Increase in physical activity and improvement of dietary quality	(124)

150 pregnant women, BMI ≤ 29 kg/m^2^, before 20 weeks gestation	Randomization to three intervention groups: healthy eating (HE), physical activity (PA), and combined healthy eating and physical activity, following principles of motivational interviewing	No significant differences in GDM risk. Lower gestational weight gain in HE compared to PA	Comparable HOMA indices in all three intervention groups. Fasting glucose lower in HE at 35–37 weeks compared to PA. 20% of all women under the weight gain target of <5 kg, in total after 37 weeks 32% GDM in obese pregnant women	(125)

436 pregnant women, BMI ≤ 29 kg/m^2^, before 20 weeks gestation	Four groups, healthy eating (HE), physical activity (PA), combined healthy eating and physical activity (HE + PA), usual care (UC), following principles of motivational interviewing	No significant differences in GDM risk. Lower gestational weight gain in HE + PA compared to UC	No Improvements in glucose or insulin parameters or HOMA IR. Similar birth weight, LGA, or SGA rates	(126)

450 pregnant women, BMI ≥ 35 kg/m^2^, 12–18 weeks gestation	Metformin, at a dose of 3.0 g per day, or placebo	No significant differences in GDM risk.^a^ Lower maternal weight gain^a^	No significant difference in birth weight. Lower incidence of preeclampsia. Higher incidence of side effects in Metformin group	(127)

449 pregnant women, BMI ≥ 30 kg/m^2^, 12–16 weeks gestation	Metformin, at a dose of 2.5 g per day (maximum dose), or placebo	No significant differences in GDM risk.^a^ No significant difference in maternal weight gain^a^	No significant difference in birth weight. No significant differences in HOMA IR or other metabolic parameters.^a^ No significant differences in combined adverse outcomes. Higher incidence of side effects in Metformin group	(128)

*^a^Secondary outcomes*.

*RCT, randomized controlled trial; BMI, body mass index; GDM, gestational diabetes mellitus; HE, healthy eating; PA, physical activity; IR, insulin resistance; RR, relative risk; CI, confidence interval; NICU, neonatal intensive care unit; LGA, large for gestational age; SGA, small for gestational age*.

A subgroup analysis of women with prediabetes at baseline included in the DPP and followed over 10 years demonstrated that women with a history of GDM have a 48% higher diabetes progression risk than women with normal glucose tolerance during pregnancy (129). In addition, intensive lifestyle changes reduced diabetes risk by 35% and metformin therapy by 40% in women with prior GDM, while in the comparison group only lifestyle intervention resulted in a risk reduction of 30% (129). This clearly shows the effectiveness of lifestyle intervention to prevent T2DM progression in female high-risk collectives, such as women with GDM. Accordingly, lifestyle modification might be a potential approach to prevent GDM progression already in pregnancy.

However, evidence of successful lifestyle intervention in pregnancy is conflicting. RCTs investigating the effects of lifestyle interventions on GDM prevention in high risk overweight/obese pregnant women yield controversial results (Table 2). Recent large scale RCTs were not able to show any significant reductions of GDM prevalence or macrosomia in offspring in the intervention groups. These studies included predominately obese pregnant women. Difficulties in motivation to participate in these studies were reported with inclusion rates of 20% of the invited women (123). Furthermore, adherence to maintain lifestyle changes throughout pregnancy was difficult. The small differences observed in weight gain in pregnancy between intervention and control groups may explain the lack of success in reduction of GDM or LGA risk (130).

Although the recently published DALI lifestyle study, investigating the effectiveness of physical activity and/or dietary intervention in decreasing GDM risk, was able to show significantly lower weight gain in the combined healthy eating and physical activity intervention group compared to usual care, there was no difference between intervention and usual care groups in glucose or insulin parameters, insulin resistance or birthweight (126).

The authors of a recent review, evaluating lifestyle intervention, dietary supplementation or pharmacological therapy for GDM prevention in women with and without risk factors for GDM, reported difficulties to give recommendations due to the complexity and heterogeneity of these trials (131). Neither combined (diet and exercise) lifestyle nor dietary interventions were able to reduce GDM progression in women without risk factors and an unclear evidence was described for physical activity. However, in obese women, dietary measures reduced GDM risk and macrosomia, but physical activity was not effective. Interestingly, supplementation with probiotics or myoinositol reduced GDM risk. Of note, pharmacological approaches, as shown for metformin, were not able to reduce GDM incidence in obese women or women with PCOS (131).

Therefore, initiation of lifestyle modification in early pregnancy might be too late. This is supported by a meta-analysis targeting increased physical activity before and during pregnancy (132). Much greater benefit was shown in studies starting intervention before pregnancy than in early pregnancy.

A recently presented large retrospective study at the American Society for Reproductive Medicine Scientific Congress including more than 78,000 women demonstrated that women with infertility have higher all-cause mortality compared to fertile women (133). Interestingly, diabetes was identified as a main primary cause of mortality, although similar diabetes prevalence in both groups was found.

A prospective cohort analysis of postmenopausal women between 50 and 79 years of age of the Women’s Health initiative revealed an association between the development of T2DM and short (less than 30 years) and long (more than 45 years) reproductive periods determined as duration from menarche to last menstrual period (134). Interestingly, HRT after menopause reduces the risk of development of T2DM and improves glycemic control in women with T2DM (118). Improvements in insulin secretion and sensitivity as well as beta cell function were reported. However, the physiological mechanisms remain elusive. Taking the existing evidence into account, the authors conclude that hormone replacement therapy within 10 years after menopause and below 60 years of age is effective in preventing development of T2DM and cardiovascular disease. Nonetheless, adverse effects of estrogen therapy need to be considered carefully before initiation.

Key points: hormonal imbalance of sex hormones and reproductive disorders, such as PCOS in women or ED in men, are associated with higher T2DM risk. Lifestyle interventions may improve hormonal imbalance and are also associated with improved cardiometabolic parameters in ED, hypogonadism and PCOS, whereas lifestyle interventions starting in early pregnancy did not affect GDM risk or pregnancy outcomes in most studies. Improvements in components of the metabolic syndrome and glycemic control were found after TRT and HRT. Myoinositol supplementation was associated with lower GDM risk and better glycemic control in women with PCOS, whereas metformin did not improve metabolic parameters in obese women with GDM or women with PCOS (Table 1).

## Recommendations for Clinical Practice and Future Research

In men and women, obesity and sedentary lifestyle are major risk factors for T2DM and weight reduction, maintenance of normal weight and physical fitness can effectively reduce the risk of diabetes. This needs to start early in life. However, many patients at risk have problems to participate in and adhere to lifestyle intervention. Same-sex-group-based exercise programs based on a social identity approach may be more successful, probably due to better motivation, adherence behavior and social congruence, but implementations and success need to be further investigated. This also applies for culturally tailored approaches. Sex-specific and culturally tailored initiatives may be very promising in tackling obesity. They could reach a great number and specific subgroups of males and females who are currently underrepresented in common gender-integrated weight loss programs. A group of special interest are Asian and Asian Americans, who should be screened for T2DM at BMI 23 kg/m^2^, which is lower than for other ethnical groups (8).

Results of several projects targeting obese and overweight male sports fans are very promising, and further examples are expected in the next few years. Indeed, there is also a need for such programs for women and the ideal setting to meet these women and engage them into a lifestyle and weight reduction program need to be identified. Although in Scandinavian countries, “Football Fitness” was able to reach a large number of women at risk, even on a population scale with the high potential to improve health outcome, this approach might not be feasible in other regions and cultures across the world. As several other aspects—such as parenting—are relevant in women’s lives potential programs also need to address these demands. Nonetheless, most programs so far were not directly addressed to investigate T2DM prevention, and therefore, studies aiming to investigate prevention of T2DM in men and women with different sociocultural and ethnical background are urgently needed.

In both sexes, reproductive factors and reproductive history may help to identify better those subjects at higher risk, such as women with early menarche, irregular cycles, PCOS, or women with a history of GDM. In males, ED can help to characterize a group at high risk. In the ADA guidelines, the importance of sex-specific aspects in diabetes development was addressed, and sex-specific differences were included in the “Diabetes Risk Test” (8). Male sex in general and women with a history of GDM are associated with higher diabetes risk. Except for GDM, information about the effectiveness of lifestyle intervention or pharmaceutical approaches in the prevention of T2DM in women with PCOS or men with ED is limited and further studies are needed.

Currently, mostly women are willing to perform bariatric surgery to reduce weight and significantly higher revision surgeries are reported to be performed in women. Data point to a higher mortality risk in men, which urgently needs closer investigation, but probably a higher age, BMI at surgery or comorbidities are potential risk factors. Sex differences are reported in complication following bariatric procedures. RYGB was associated with a good outcome regarding weight loss and several other parameters in both sexes, whereas gastric banding seems to be less efficient in men. Furthermore, male sex is often mentioned in literature to be associated with higher complication rate and worse follow-up outcome after bariatric surgery. Lower weight loss after RYGB, sleeve gastrectomy, and gastric banding was reported in African American compared with Hispanic and non-Hispanic white patients.

Although trials investigating lifestyle or pharmacological intervention in males and females at risk were promising so far, there is need of more research on biological and psychosocial differences and of evidence-based sex-sensitive and culturally tailored concepts of prevention. However, so far most published study results are derived from secondary *post hoc* analysis and thus are mostly insufficiently powered. Thus, further prevention trials are necessary to investigate sex, gender, and ethnical differences in effectiveness of prevention strategies and short- and long-term health outcomes, such as cardiovascular endpoints. Moreover, adverse events and potential sex-specific differences need to be reported in prevention trials using glucose lowering compounds. They are widely not documented and thus essential information is still missing.

In summary, future studies, should consider and investigate sex and gender as well as ethnical differences in diabetes and cardiovascular prevention trials to a much higher extent than so far performed. The consideration and implementation of sex/gender differences in clinical practice and in diagnostic and therapeutic concepts—if necessary—will improve quality of care and reduce the burden of growing diabetes numbers. This considerably will lower health care costs worldwide. Sex-sensitive and culturally tailored prevention programs and sex-specific education, lifestyle programs, and drug therapy will potentially contribute to better care of patients with T2DM in the future.

## Author Contributions

JH and AK-W contributed to conception or design, acquisition, analysis, or interpretation of the manuscript. JH and AK-W drafted and critically revised the manuscript. JH and AK-W gave final approval and agreed to be accountable for all aspects of work ensuring integrity and accuracy.

## Conflict of Interest Statement

The authors declare that the research was conducted in the absence of any commercial or financial relationships that could be construed as a potential conflict of interest.
